# Enhancement of the catalytic performance of Co-ZIF/WO_3_ heterostructures for selective catalytic reduction of NO_x_

**DOI:** 10.1038/s41598-024-53805-7

**Published:** 2024-02-08

**Authors:** Hassan Alamgholiloo, Esrafil Asgari, Amir Sheikhmohammadi, Naser Ghasemian, Bayram Hashemzadeh, Heshmatollah Nourmoradi

**Affiliations:** 1 Department of Environmental Health Engineering, School of Health, Khoy University of Medical Sciences, Khoy, Iran; 2 Department of Chemical Engineering, University of Bonab, Bonab, Iran; 3 Health and Environment Research Center, Ilam University of Medical Sciences, Ilam, Iran; 4 Department of Environmental Health Engineering, School of Health, Ilam University of Medical Sciences, Ilam, Iran

**Keywords:** SCR-NOx, Propane, Heterostructure, Co-ZIF/WO_3_, Environmental sciences, Natural hazards, Chemistry

## Abstract

Nitrogen oxides (NOx) are one of the growing air pollutants in industrial countries, and their emissions are regulated by stringent legislation. Therefore, the design of the catalyst comprised of metal oxides and ZIFs a potential solution for improving selective catalytic reduction (SCR) of NOx. Here, an efficient strategy was described to fabricate Co-ZIF/WO_3_ heterostructures for SCR of NOx. First, WO_3_ nanostructures were fabricated by the solvothermal method, and subsequently epitaxial growth of ZIF-67 on the metal oxide surface to create a new type of semiconductor Co-ZIF/WO_3_ heterostructures. The obtained heterostructures were systemically characterized by wide-angle XRD, FESEM, UV DRS, FT-IR, AFM, and TEM spectroscopies. The Co-ZIF/WO_3_ heterostructures shift the temperature corresponding to the maximum conversion around 50 °C towards lower temperatures. The maximum conversion is substantially enhanced from 55% at 400 °C to 78% at 350 °C. The enhanced activity is attributed to better interaction and synergic effect of WO_3_ incorporated into ZIF-67 and also the electron transfer facility between the WO_3_ and Co species in Co-ZIF/WO_3_ heterostructures. Moreover, Co-ZIF/WO_3_ results in a distinct effect on the production of carbon monoxide (CO) in the product gas stream. The current study highlights some of the challenges in the development of semiconductor-based heterostructures for a decrease in air pollution.

## Introduction

Combustion of fossil fuels and burning of biomass has caused air pollution and environmental, creating health problems in the world^1–4^. Therefore, the issue of air pollution has seriously affected people's ability to have a better life^2,5^. Nitrogen oxides (NO_2_ + NO = NOx) as one of the air pollutants play a vital role in tropospheric chemistry such as ozone formation, production of secondary aerosols, and acid rain^6,7^. NOx pollutants are increasingly produced from a variety of human activities and natural resources^7^. Among NOx gases, NO_2_ has the highest concentration in the ambient air and can be fatal if inhaled in large quantities^8^. Furthermore, this poisonous gas has a reddish-brown color with a boiling point of 21.1 °C and low partial pressure that keeps it gaseous^8,9^. Also, this corrosive gas has a strong oxidant and physiologically stimulates the lower respiratory tract and thereby is toxic to humans^9^. Most atmospheric NO_2_ is released as NO, which is rapidly oxidized by ozone to NO_2_. Also, NOx gases can combine with common organic matter and even ozone to form a wide range of toxic compounds, which can cause mutations in DNA^10,11^. In addition, NOx can cause serious respiratory problems, odor disorders, fatigue, throat irritation, nerve disorders, and increased acute bronchitis in humans^12,13^. Consequently, developing an efficient method for the complete removal of NOx has become a global priority.

Recently, different methods have been expanded to control these gaseous pollutants, including thermal^14^, adsorption^15^, and catalytic^16,17^ methods due to the importance of this pollutant in the environment. In the thermal method, a high temperature is used to decompose NO, which high temperature causes undesirable side reactions^14^. In the adsorption method, the efficiency of the adsorbent is reduced due to the adsorption of NOx on its active surface^18^. Therefore, these two methods are not suitable for NOx removal. Recently, many investigate have reported the efficient method of selective catalytic reduction (SCR) for NOx abatement via different hydrocarbon compounds such as propane^19,20^, methane^21^, and ammoniac as a reductant. Moreover, semiconductor materials with different charge migration pathways have been used in an industrial SCR system^22^. WO_3_-based materials as efficient semiconductors have been considered to be a promising photocatalyst in abating NOx emissions from air pollution^23^. Unfortunately, the low electrical conductivity and negligible specific surface area have restricted the performance of pure WO_3_ in the SCR-NOx-Propane process. Fortunately, the conjugation of this semiconductor material with Zeolites could improve light harvesting and charge separation for the removal of NOx^24^. Zeolites such as ZSM-5^25^, clinoptilolite^26^, and metal oxides including cerium oxide^27^, zirconium oxide^28^, vanadium oxide^29^, and tungsten oxide^23^ have been proposed as catalysts for the removal of NO_x_ with various methodologies. Recently, Lee et al. developed CuSn/ZSM-5 for the HC-SCR-NOx process^30^. Moreover, the effect of the addition of molybdenum on the enhanced low-temperature SCR of NOx by NH_3_ over MnOx/γ-Al_2_O_3_ catalysts was investigated by Yang and et al.^31^. Furthermore, Zhan et al. reported mesoporous WO_3_ for SCR-NOx with NH_3_^32^. Recently, our research team developed engineered nanostructures to expand the photo-based advanced oxidation process (AOP)^33–36^ and persulfate-based AOP^4,37–39^ for the removal of organic contaminations from wastewater.

Zeolitic imidazolate frameworks (ZIFs) with the advantages of uniform pore size and high BET surface have been used as catalysts for selective catalytic reductions (SCR)^40,41^. Recently, ZIF-based materials like ZIF-67 have been utilized as an efficient microporous for NO_x_ abatement and its performance in the SCR-NOx process has been investigated^42^. Recently, Zhao et al. reported Cu-ZIF performance on NH_3_-SCR-NOx^42^. Moreover, the performance of Co_3_O_4_-PC derived from ZIF-67 for low-temperature SCR of NOx by ammonia was studied by Bai et al.^42^. However, the activity of ZIF-67 on propane-SCR-NOx has not been studied so far. Therefore, the activity of ZIF 67 as an efficient catalyst for NOx abetment is investigated. In this study, we have attempted to develop an efficient nanostructure for promoting the activity of WO_3_ as a conventional catalyst for NOx abatement. Accordingly, a porous nanocomposite of Co-ZIF/WO_3_ due to the better interaction and synergic effect of WO_3_ nanostructure incorporated into ZIF-67 was used. Also, the electron transfer facility between the WO_3_ and Co species in the channels of Co-ZIF/WO_3_ exhibited a lower energy band gap of Co-ZIF/WO_3_ led to enhancing the catalytic activity of Co-ZIF/WO_3_ in the NOx process. Therefore, a part of the study belongs to the design, fabrication, and characterization of nanocomposite of Co-ZIF/WO_3_ heterostructure and another part belongs to the performance of WO_3_, ZIF-67, and Co-ZIF/WO_3_ in conversion and temperature on propane-SCR-NOx. According to the latest findings reported, there is no study on the design of Co-ZIF/WO_3_ nanocomposite for selective catalytic reduction (SCR) of NOx. Therefore, the current study provided a rational design for a decrease in air pollution.

## Experimental

### Material

Tungstate dihydrate (Na_2_WO_4_·2H_2_O), cobalt nitrate hexahydrate (Co(NO_3_)_2_·6H_2_O), 2-methylimidazole (MeIM), polyvinylpyrrolidone (PVP), oxalic acid (C_2_H_2_O_4_) were purchased from Sigma-Aldrich Co (USA) and and Merck Co. (Germany). Furthermore, hydrochloric acid (HCl), methanol (MeOH), ethanol (EtOH), and other solvents were used without further purification.

### Characterization

The characterization of as-prepared nanostructures and catalytic reduction of NO_x_ is described in text S1 of supplementry Information.

### Fabrication of WO_3_ nanoplates

The WO_3_ nanostructure was prepared following a method described by Zheng and co-workers^43^. In a typical experiment, 8.25 g Na_2_WO_4_·2H_2_O was dissolved in 25 mL dionized water under sonication. Subsequently, 2.0 mL of 2.0 M HCl was added to the mixture reaction and then oxalic acid was added to the mixture to adjust the final pH value to approximately 2.50 and diluted to 250 mL. After that, 1.17 g NaCl was added to the mixture reaction under sonication. After 10 min sonication, 70 mL of the above precursor solution was transferred into a 100 mL of Teflon-lined autoclave and heated for 4.0 h at 170 °C. After completion of the reaction, the resulting yellow precipitate was washed several times with DI-H_2_O and EtOH for purification to obtain the final WO_3_ nanoplates.

### Fabrication of Co-ZIF/WO_3_ heterostructure

**Figure 1:**
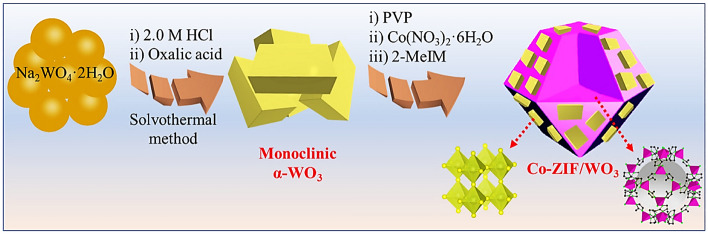
Synthetic strategy for assembly of Co-ZIF/WO_3_ heterostructures.

To fabricate Co-ZIF/WO_3_ heterostructures, firstly 0.20 g of as-synthesized WO_3_ with 2.0 mmol PVP as structure-director agents was dispersed in 100 mL MeOH under sonication. Subsequently, 1.05 g of Co(NO_3_)_2_·6H_2_O was added to the mixture reaction shaken vigorously for 15 min. Afterward, 6.15 g 2-MeIM was added to the mixture reaction under stirring for 24 h to grow ZIF-67 microcrystals on the WO_3_ surface. Finally, the prepared precipitates were separated by centrifugation and washed with MeOH several times, and dried at 80 °C under a vacuum. The schematic fabrication of the Co-ZIF/WO_3_ heterostructure is revealed in Fig. 1.

### Investigation of catalytic activity

**Figure 2:**
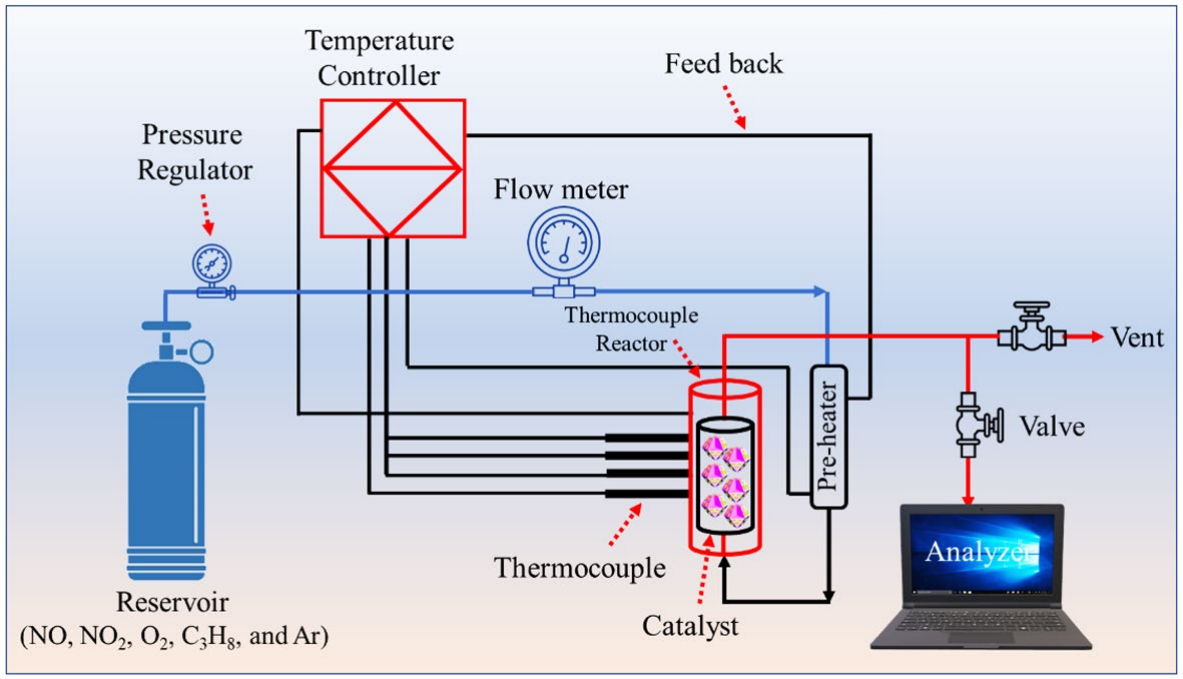
Catalytic reactor for the SCR-NO_x_ process in the study.

The schematic catalytic reactor system used in this study was displayed in Fig. 2. In brief, the inlet gas mixture involved nitric oxide (30 mg/L), nitrogen dioxide (460 mg/L), oxygen (2.5 vol%), C_3_H_8_ (1.0 g/L), and also argon gas (balance) was introduced to the flow meter set at 300 mL/min. Afterward, the gas mixture was preheated and also conducted in an integral reactor containing 0.50 g of the catalyst. A stainless steel vessel was used as a reactor with a diameter of 0.50 inches. An electrical furnace was applied to heat the reactor. Various thermocouples at the inlet, inside the reactor bed, and outlet of the stream were employed to control system temperature. This system controlled the temperature of the reactor bed over 150–400 ± 1 °C. Gas analysis of the outlet was measured using a sensor probe. Finally, the KANE 940 gas analyzer was conducted to evaluate gases of NO, NO_2_, O_2_, and CO.

## Result and discussion

**Figure 3:**
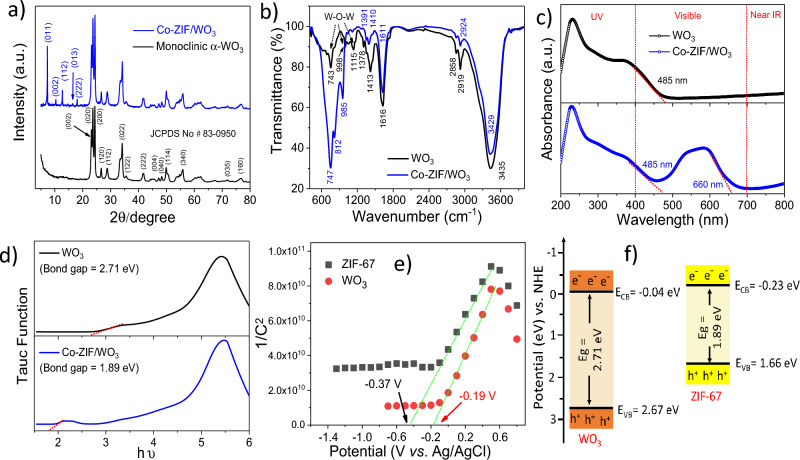
The HA-XRD pattern (**a**), FT-IR spectra (**b**), UV–Vis DRS spectrum (**c**), Tauc plot (**d**), Mott-Schottky plot (**e**), and bandgap structures of obtained nanostructures.

The crystalline phase of WO_3_ and Co-ZIF/WO_3_ was assessed using the HA–XRD pattern. As depicted in Fig. 3a, the characteristic diffraction peaks at 22.1°, 23.6°, 24.3°, 34.1°, 41.7°, 49.8°, 54.9°, and 55.8° be indexed α-WO_3_ with monoclinic phase and JCPDS Card No. 83-0950^36^. After epitaxial growth of ZIF-67 on surface WO_3_, all peaks related to crystal ZIF-67 were revealed in ZIF-67/WO_3_ heterostructure^38,39^. Moreover, the chemical groups of the heterostructure were evaluated by FT-IR spectra (Fig. 3b). The peaks at 600–800 cm^−1^ can be ascribed to W–O–W stretching vibration^44^, confirming the formation of WO_3_. The weak peaks at 460 cm^−1^ revealed the vibration of the Co–N, respectively^45–47^ and bands in the range of 800–1300 corresponded to the symmetric and asymmetric stretching of the imidazole rings^47^, confirming the formation of ZIF-67 on WO_3_ surface. The optical response characteristics of as-obtained nanostructures were evaluated by UV–Vis DRS spectra. As shown in Fig. 3c, the absorption edge of WO_3_ was approximately 470 nm, while after epitaxial growth of ZIF-67 on WO_3_ exhibited a strong absorption intensity from 350 to 650 nm. Meanwhile, the broad absorption in the UV region (< 400 nm) can be attributed to ligand to metal charge-transfer (LMCT)^36,48^, while displaying three absorption peaks at 538, 565, and 590 nm indexed to the ^4^A_2_(F) → ^4^T_1_(P) transition of Co^2+^ ions in ZIF-67 framework^48–50^. Also, the band-gap values for WO_3_ and ZIF-67/WO_3_ heterostructure were 2.71 and 1.89 eV, respectively (Fig. 3d), indicating an increase in light absorption capacity. As depicted in Fig. 3e, Co-ZIF and WO_3_ revealed positive slopes in the linear regions of the Mott-Schottky plots, illustrating both the nanostructures have n-type semiconductor behavior. Meanwhile, the flat band potential (E_fb_) derived from Mott-Schottky plots of WO_3_ and Co-ZIF were approximately − 0.19 and − 0.37 V (vs Ag/AgCl, pH 7), which are equivalent to − 0.04 V and − 0.23 (vs NHE, pH 0) Eq. (1) ^36^. According to (Eqs. (1)–(3) ^36^ and Mott-Schottky plot, the conduction band (CB) and valence band (VB) of WO_3_ were calculated to be − 0.04 eV and 2.67 eV, while those of Co-ZIF were obtained to be − 0.23 eV and 1.66 eV, respectively (Fig. 3f).

1 $$ {\text{E}}_{{\left( {{\text{Vs}}.{\text{ NHE}},{\text{ pH }}0} \right)}} = {\text{ E}}_{{({\text{Vs}}.{\text{ Ag}}/{\text{AgCl}},{\text{ pH 7}})}} - \, 0.0{591 }\left( {{7 }{-}{\text{ pH}}_{{{\text{electrolyte}}}} } \right) \, + \, 0.{198} $$

2 $$ {\text{E}}_{{{\text{VB}}}} = {\text{ E}}_{{{\text{CB}}}} + {\text{ E}}_{{\text{g}}} $$

3 $$ {\text{E}}_{{{\text{CB}}}} = {\text{ E}}_{{({\text{Ag}}/{\text{AgCl}})}} + {\text{ E}}^{\theta }_{{({\text{Ag}}/{\text{AgCl}})}} + \, 0.0{\text{591 pH}} $$

**Figure 4:**
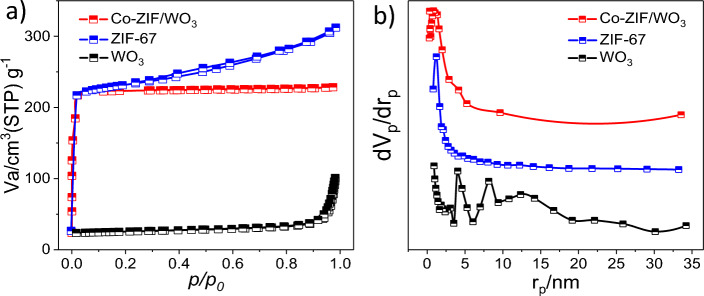
N_2_ adsorption–desorption isotherms (**a**) and BJH plot (**b**) for Co-ZIF/WO_3_, ZIF-67, and WO_3_ nanostructures.

Moreover, N_2_ adsorption–desorption isotherm was conducted for the evaluation of the surface area and pore size of Co-ZIF/WO_3_ heterostructure. As depicted in Fig. 4a and Table 1, the heterostructure indicated a typical type I isotherm with surface area S_BET_ = 1061 m^2^/g and total pore = 0.35 cm^3^/g. Meanwhile, ZIF-67 and WO_3_ revealed a typical type II and III isotherms with S_BET_ = 1420 m^2^/g and 16.17 m^2^/g, respectively. According to the BJH plot (Fig. 4b), the corresponding pores diameter distributions of the Co-ZIF/WO_3_, ZIF-67, and WO_3_ were specified at 1.21, 1.23, and 4.69 nm respectively. These microporous structures with larger specific surface areas in Co-ZIF/WO_3_ heterostructure can be provided suitable to the adsorption of gases and catalytic activity.

**Table 1:** Textural properties of samples.

Samples	Textural properties		
	BET (m^2^/g)	Pore diameter (nm)	Pore volume (cm^3^/g)
WO_3_	16.176	4.69	0.119
ZIF-67	1420.34	1.23	0.734
Co-ZIF/WO_3_	1061.51	1.21	0.351

**Figure 5:**
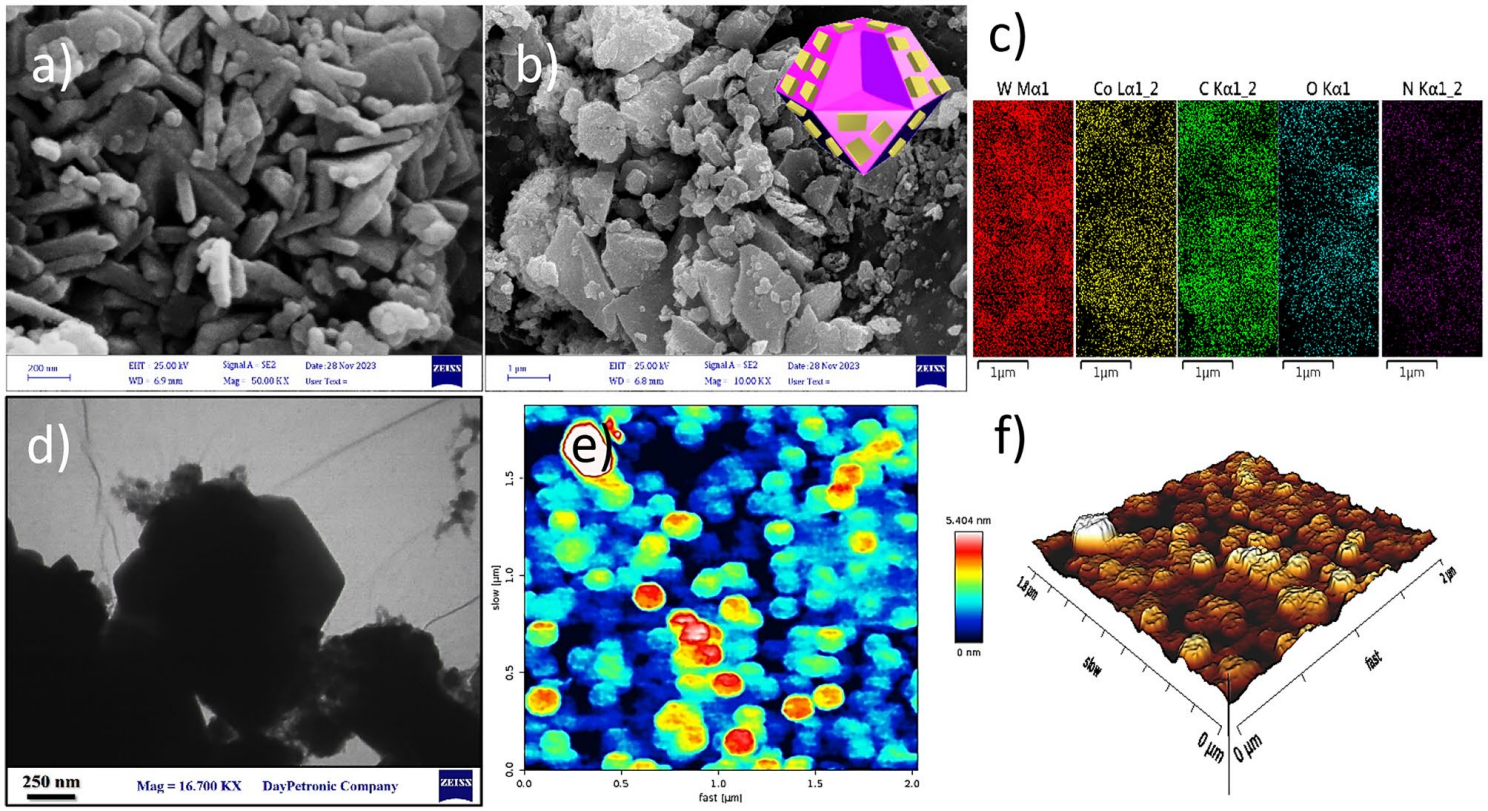
FESEM images of WO_3_ (**a**) and Co-ZIF/WO_3_ (**b**); FESEM-mapping of Co-ZIF/WO_3_ (**c**); TEM image of Co-ZIF/WO_3_ (**d**); and 2D AFM and 3D AFM images of Co-ZIF/WO_3_ (**e**, **f**).

The morphology and structure of the prepared catalysts were evaluated by FESEM, AFM, and TEM. The pure WO_3_ exhibited a plate-like structure with a thickness of about 45 nm (Fig. 5a). After the growth of the ZIF-67 on WO_3_, these metal oxides were heterogeneously dispersed over the surface of ZIF-67 (Fig. 5b). Meanwhile, the structures and morphology of ZIF-67 and WO_3_ about preserved after composition. Also, SEM-mapping (Fig. 5c) and EDS analysis (Fig. S1, ESI) demonstrated the purity and elements corresponding to the heterostructure. Moreover, the presence of WO_3_ nanoplates in the core of the proposed heterostructure was not observed with FESEM images; therefore, the TEM image was conducted to further evaluated the morphology and growth of ZIF-67 with WO_3_. As displayed in Fig. 5d, WO_3_ nanoplates were observed on the surface of ZIF-67 with rhombic dodecahedral framework morphology. This image further confirmed the formation of Co-ZIF/WO_3_ to composite form. Furthermore, AFM analysis indicated surface morphology and roughness of the proposed heterostructure. As depicted in Fig. 5e and f, the arithmetic average roughness (Ra) of Co-ZIF/WO_3_ was approximately 150 nm, which appeared in light and dark regions in the 2D and 3D images.

**Figure 6:**
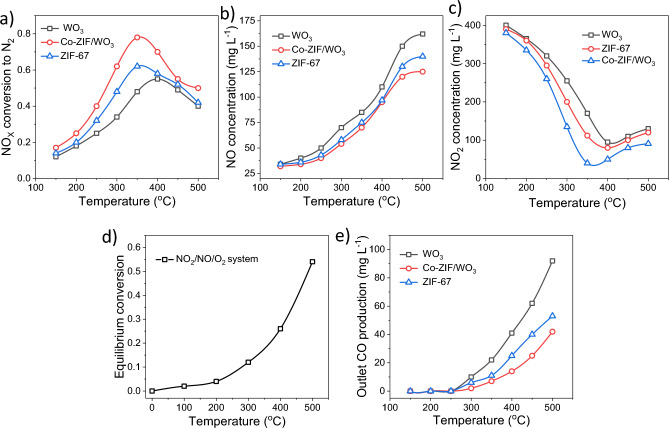
The effect of the reaction temperature and kind of sample on the conversion of NO_x_ (NO + NO_2_) into N_2_ (**a**), the variation of NO concentration (**b**), the variation of NO_2_ concentration in the exhaust gas for different nanostructures (**c**), The equilibrium conversion of NO_2_ to NO in the three component system NO_2_–NO–O_2_ (**d**), and outlet CO concentration of production versus reaction temperature for all samples (**e**).

After characterization of the proposed heterostructure, the activity of WO_3_, ZIF-67, and Co-ZIF/WO_3_ was evaluated on the conversion of NOx to N_2_. The total NOx to N_2_ conversion curve as a function of reaction temperature for all samples at gas hourly space velocity (GHSV) 51,048 h^−1^. As depicted in Fig. 6a, all samples reveal a similar trend in NOx reduction efficiency. Meanwhile, all samples have a maximum conversion at 350–400 °C, then activity was decreased. The smallest conversion factor (55%) belongs to WO_3_ nanoplates at 400 °C due to not being porous material and having a low specific surface area. Also, the maximum activity occurred for ZIF-67 and Co-ZIF/WO_3_ heterostructure with a conversion rate of 62% and 78% at 350 °C, respectively. Therefore, the corresponding maximum transition temperature changes from 50 °C to a lower temperature for ZIF-67 and Co-ZIF/WO_3_ heterostructure. Research has revealed that WO_3_ is an n-type semiconductor with negligible porous structure, which has the least conversion of NOx into N_2_ in comparison to ZIF-67 and Co-ZIF/WO_3_. Meanwhile, the ZIF-67 indicated a suitable conversion of NOx into N_2_ due to the microporous structure and high surface area (S_BET_ = 1420.34 m^2^/g, Table 1 and Fig. S2, ESI). Also, ZIF-67 contains cobalt species that can promote the active site in the SCR-NOx process. Therefore, the composition of WO_3_ nanoplates with ZIF-67 microcrystals led to increasing catalytic activity due to interaction and electron transfer facility between the WO_3_ and Co species in the Co-ZIF/WO_3_. It should be noted that in some additional runs, the mass of prepared nanostructures and GHSV were increased by 20% at the constant reaction temperature. Since no significant changes in turnover were observed at the concentrations of N_2_, NO_2_, NO, and CO. It was easy to conclude that there was no resistance to mass transport of the gas layer in any of the other experiments. Figure 6b demonstrates the development of the NO_2_ concentration in the flue gases as a function of the reaction temperature in the range from 150 to 500 °C for different samples at GHSV 51,048 h^−1^. An S-shaped curve (decreasing with increasing temperature) was obtained for each sample. Particularly noteworthy is the variability of the NO concentration in the generated gas depending on the reaction conditions. Also, the change in the NO concentration of the generated gas with the reaction conditions is worth special evaluation. It is known that Mo (650 °C), Ag (160 °C), and stainless steel (450 °C) can catalyze the decomposition reaction of NO_2_ to NO. As depicted in Fig. 6b and c, Co-ZIF/WO_3_ revealed the lowest NO production concentration and the highest NO_2_ concentration reduction. It should be borne in mind that the main nitrogenous reagent in our system is NO_2_ due to the decomposition of NO_2_ into NO and O_2_ at temperatures above 200 °C. As depicted in Fig. 6d, in a three-component system NO_2_–NO–O_2_ conversions up to 50% are expected at 500 °C due to the residence time of NO_2_ species in the reaction system. The findings indicated the formation of CO as an undesired product is inevitable during the NOx reduction in presence of hydrocarbons. As displayed in Fig. 6e, the CO production concentration in Co-ZIF/WO_3_ heterostructure is a minimal value compared to WO_3_ nanoplates and ZIF-67 microporous (35 ppm at 500 °C). According to our previous studies^26,51^, this phenomenon may be related to the non-selective combustion of hydrocarbons (propane) at higher temperatures. Furthermore, the CO generation reaction can activate in Co-ZIF/WO_3_ heterostructure channel and does not exclusively take place in the gas phase. However, it is observed that the CO concentration increases with the increase of the reaction temperature, which can be justified by considering the following reactions [Eqs. (4)–(8)]^26^.

4 $$ {\text{2C}}_{{3}} {\text{H}}_{{8}} + { 1}0{\text{NO}}_{{2}} \to {\text{ 5N}}_{{2}} + {\text{ 6CO}}_{{2}} + {\text{ 8H}}_{{2}} {\text{O}} $$

5 $$ {\text{C}}_{{3}} {\text{H}}_{{8}} + {\text{ 2NO }} + {\text{ 4O}}_{{2}} \to {\text{ N}}_{{2}} + {\text{ 3CO}}_{{2}} + {\text{ 4H}}_{{2}} {\text{O}} $$

6 $$ {\text{C}}_{{3}} {\text{H}}_{{8}} + {\text{ 5O}}_{{2}} \to {\text{ 3CO}}_{{2}} + {\text{ 4H}}_{{2}} {\text{O}} $$

7 $$ {\text{2C}}_{{3}} {\text{H}}_{{8}} + {\text{ 7O}}_{{2}} \to {\text{ 6CO }} + {\text{ 8H}}_{{2}} {\text{O}} $$

8 $$ {\text{2NO}}_{{2}} \leftrightarrow {\text{ 2NO }} + {\text{ O}}_{{2}} $$

## Conclusion

The present study was planned to design and fabricate Co-ZIF/WO_3_ heterostructure for NOx reduction emissions. The results obtained from the XRD, FESEM, and TEM indicate the epitaxial growth of ZIF-67 microcrystals onto the WO_3_ nanoplates. The catalytic efficiency of the Co-ZIF/WO_3_ in terms of NOx reduction is better than that of Co-ZIF, and WO_3_. This enhanced activity is attributed to the synergic effect of WO_3_ nanoplates loaded into ZIF-67 microcrystals and also the electron transfer facility between them in Co-ZIF/WO3 heterostructures. Moreover, the outlet concentration of CO is lower for Co-ZIF/WO_3_ than for ZIF-67, and WO_3_, which is an undesirable product in the SCR-NOx-Propane process. Our future efforts will be devoted to designing a new type of nanostructure for the reduction of the CO concentration to near 5 ppm. Finally, our findings provide a feasible strategy for the development of semiconductor-based heterostructures for reducing NOx emissions.

### Supplementary Information

Supplementary Information.

## Data Availability

The datasets used and/or analyzed in this study are available in the manuscript can be asked from the corresponding author upon request.
