# Supplementary material for: Enhancement of the catalytic performance of Co-ZIF/WO3 heterostructures for selective catalytic reduction of NOx
Source: Sci Rep. 2024 Feb 8;14:3277. doi: 10.1038/s41598-024-53805-7 (PMC10853505; doi:10.1038/s41598-024-53805-7)
Supplement: Supplementary file 1 — Supplementary Information. [file 41598_2024_53805_MOESM1_ESM.docx]

**Electronic Support Information**

**Enhancement the catalytic performance of Co-ZIF/WO_3_ heterostructures for selective catalytic reduction of NO_x_**

Hassan Alamgholiloo^1,^ Esrafil Asgari^1,*^, Amir Sheikhmohammadi^1,*^, Naser Ghasemian^2,*^, Bayram Hashemzadeh^1^, Heshmatollah Nourmoradi^3,4^

^1^Department of Environmental Health Engineering, School of Health, Khoy University of Medical Sciences, Khoy, Iran.

^2^Department of Chemical Engineering, University of Bonab, Bonab, Iran.

^3^Health and Environment Research Center, Ilam University of medical Sciences, Ilam, Iran.

^4^Department of Environmental Health Engineering, School of Health, Ilam University of medical Sciences, Ilam, Iran.

***Correspondence:**

Esrafil Asgari: esrafil_asgari@khoyums.ac.ir

Amir Sheikhmohammadi: [a_sheikhmohammadi@khoyums.ac.ir](mailto:a_sheikhmohammadi@khoyums.ac.ir)

Naser Ghasemian: n.ghasemian@gmail.com

**Table of Contents**

**Texts**

**Text S1.** Characterization

**Figures**

**Fig. S1.** EDS of Co-ZIF/WO_3_ heterostructure.

**Fig. S2.** BET and BJH plot of Co-ZIF-67.

**Text S1.** Characterization

The morphology and structure of the nanomaterials were carried out by field emission scanning electron microscopy (FESEM) Zeiss-SIGMA VP, and transmission electron microscopy (TEM) Zeiss-EM 900. The crystalline structure of the samples was taken by high-angle XRD (HA-XRD) with Panalytical-Pert Pro ^'^X. Atomic force microscopy (AFM) was analyzed by a Dual Scope TMDS 95–200/50 apparatus. FT-IR, PerkinElmer**-**Spectrum Two was recorded to study functional groups. The N_2_O species concentration in the effluent was analyzed with gas chromatography (SHIMADZU model 2010 plus) equipped with a Molecular sieve column system and a thermal conductivity detector (TCD).


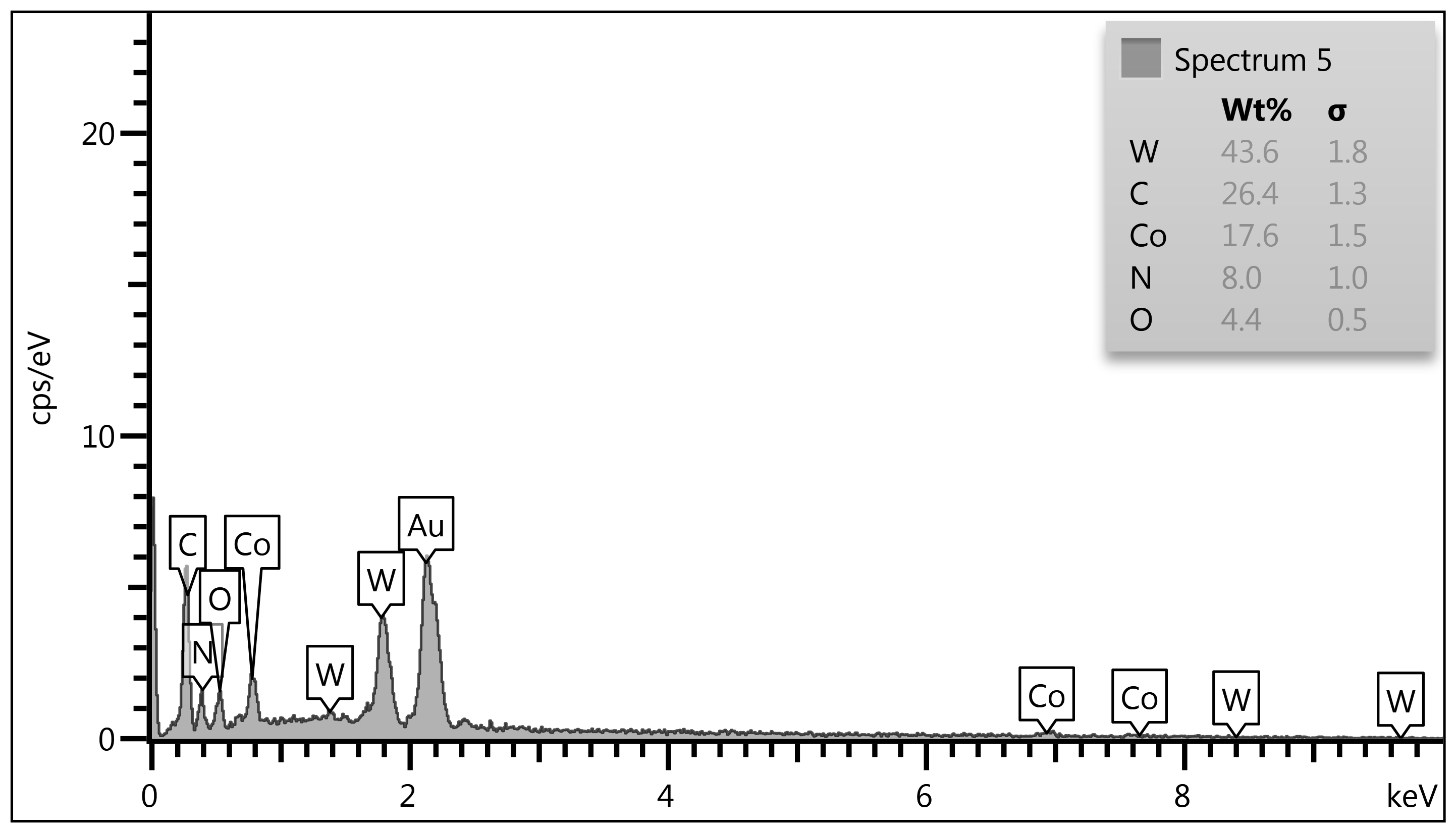


**Fig. S1.** EDS of Co-ZIF/WO_3_ heterostructure.


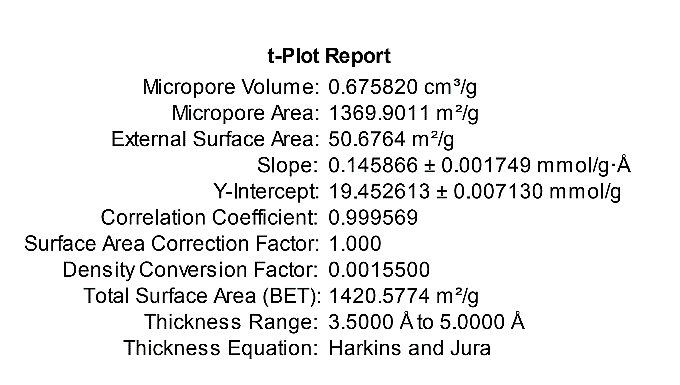

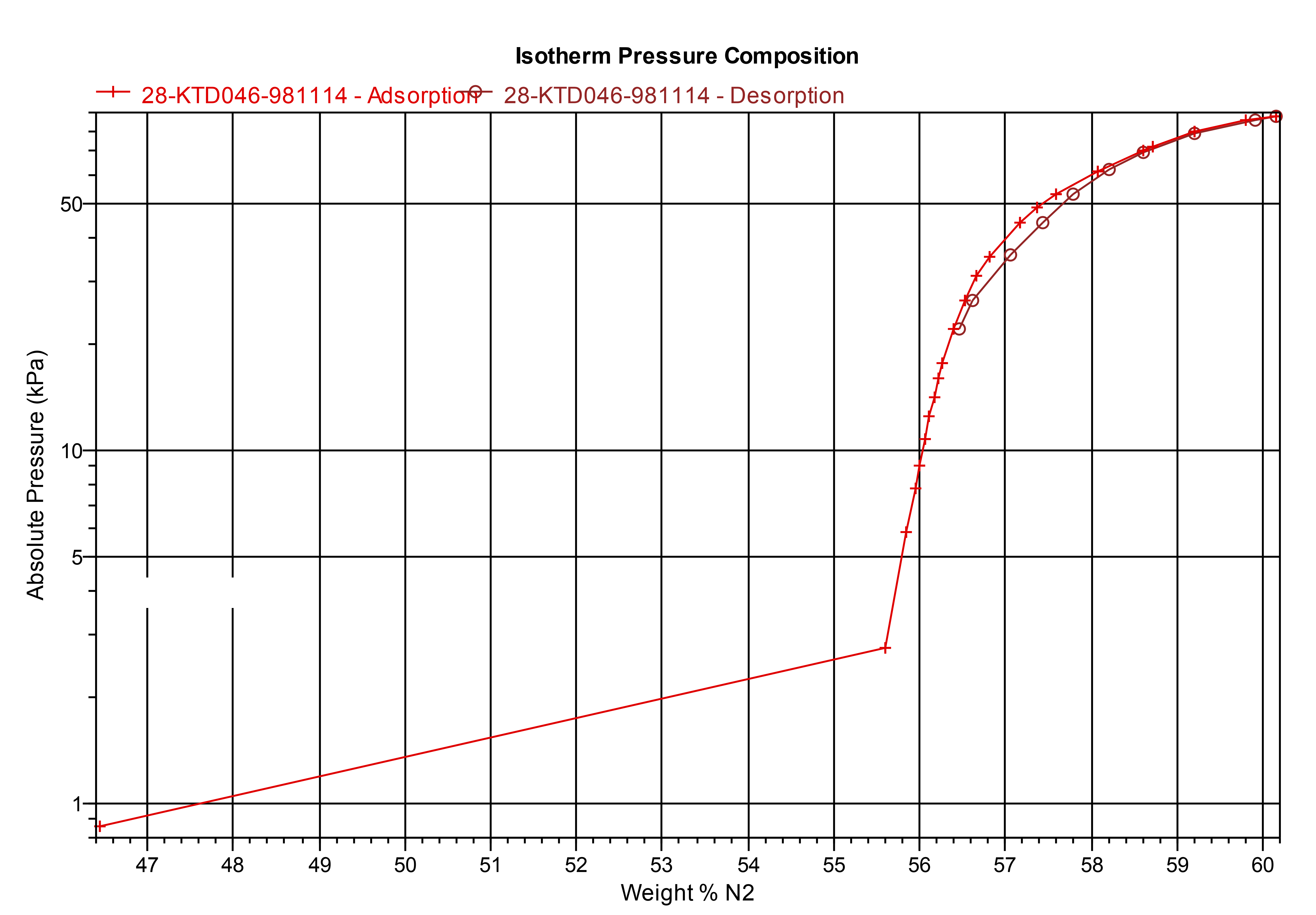


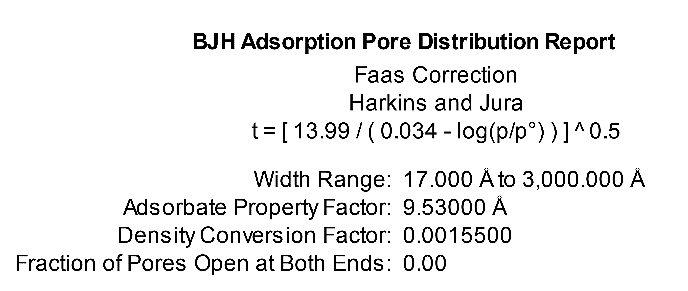

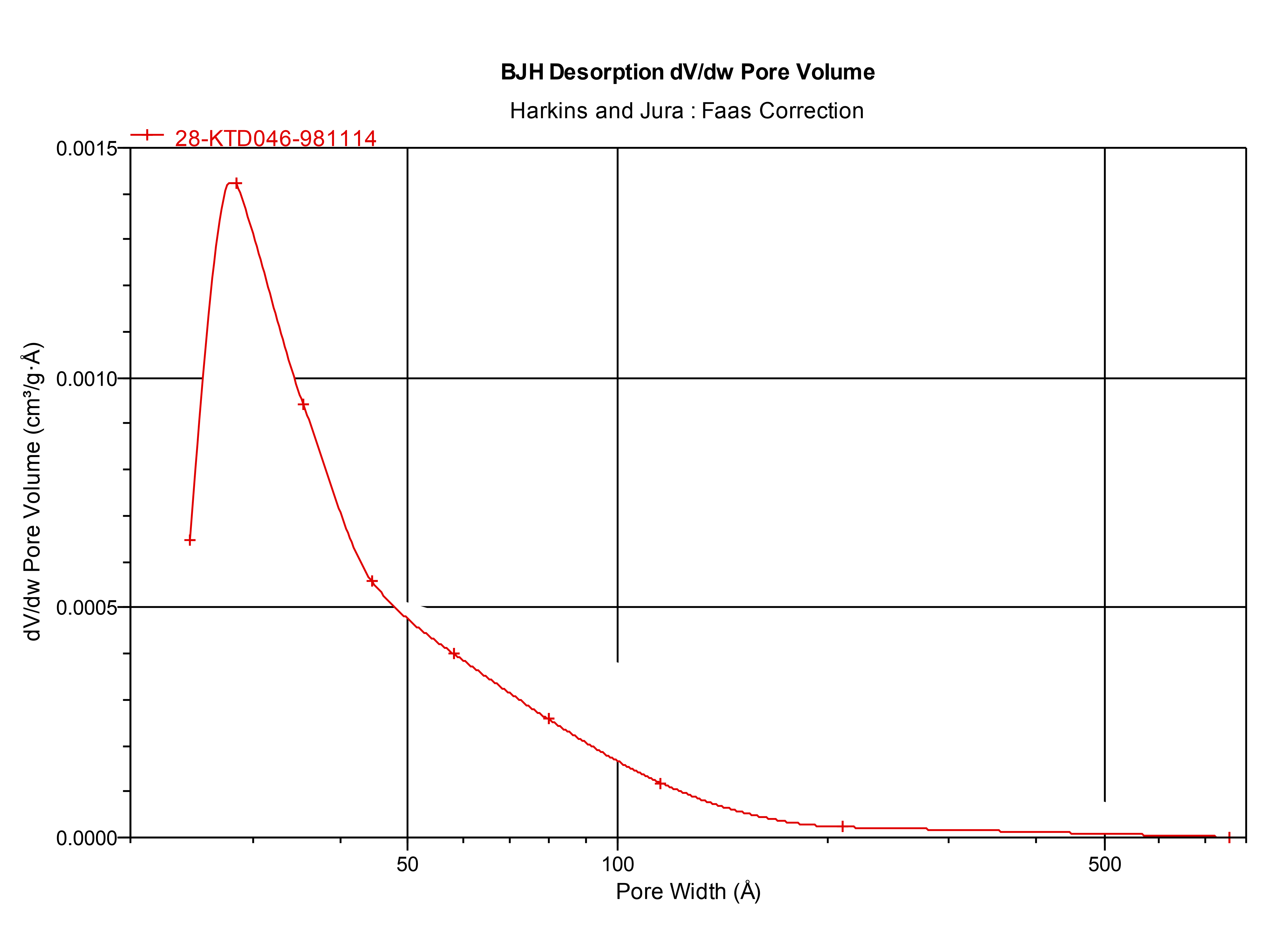


**Fig. S2.** BET and BJH plot of ZIF-67.
